# 

**Published:** 1897-05-01

**Authors:** 


CADBURY'S COCOA
"The standard of highest purity at present attainable."—*Lancet.*

CADBURY'S COCOA
NO ALKALIES USED.

### THE HOSPITAL

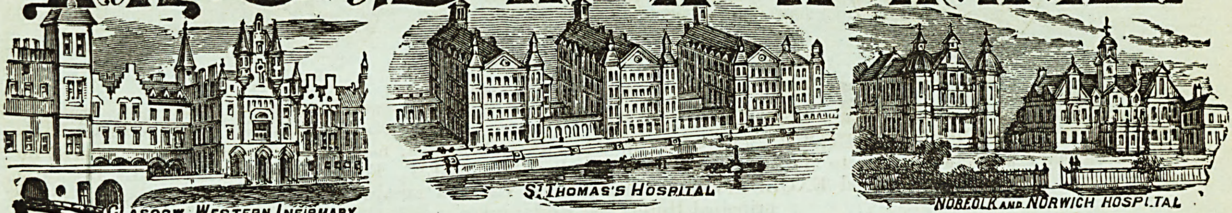

No. 553. Vol. XXII. [Registered as a Newspaper.] SATURDAY, MAY 1ST, 1897. [Subscription Terms, see p. 72.] PRICE TWOPENCE.

